# Fully automated, inline quantification of myocardial blood flow with cardiovascular magnetic resonance: repeatability of measurements in healthy subjects

**DOI:** 10.1186/s12968-018-0462-y

**Published:** 2018-07-09

**Authors:** Louise A. E. Brown, Sebastian C. Onciul, David A. Broadbent, Kerryanne Johnson, Graham J. Fent, James R. J. Foley, Pankaj Garg, Pei G. Chew, Kristopher Knott, Erica Dall’Armellina, Peter P. Swoboda, Hui Xue, John P. Greenwood, James C. Moon, Peter Kellman, Sven Plein

**Affiliations:** 1 Multidisciplinary Cardiovascular Research Centre (MCRC) & Leeds Institute of Cardiovascular and Metabolic Medicine, University of Leeds, Clarendon Way, Leeds, LS2 9JT UK; 2 Medical Physics and Engineering, Leeds Teaching Hospitals NHS Trust, Leeds, LS1 3EX UK; 3 Barts Heart Centre, The Cardiovascular Magnetic Resonance Imaging Unit and The Inherited Cardiovascular Diseases Unit, St Bartholomew’s Hospital, West Smithfield, London, UK; 4 National Heart, Lung, and Blood Institute, National Institutes of Health, DHHS, Bethesda, MD USA

**Keywords:** Myocardial perfusion, Cardiac magnetic resonance, Myocardial perfusion reserve

## Abstract

**Background:**

Non-invasive assessment of myocardial ischaemia is a cornerstone of the diagnosis of coronary artery disease. Measurement of myocardial blood flow (MBF) using positron emission tomography (PET) is the current reference standard for non-invasive quantification of myocardial ischaemia. Dynamic myocardial perfusion cardiovascular magnetic resonance (CMR) offers an alternative to PET and a recently developed method with automated inline perfusion mapping has shown good correlation of MBF values between CMR and PET. This study assessed the repeatability of myocardial perfusion mapping by CMR in healthy subjects.

**Methods:**

Forty-two healthy subjects were recruited and underwent adenosine stress and rest perfusion CMR on two visits. Scans were repeated with a minimum interval of 7 days. Intrastudy rest and stress MBF repeatability were assessed with a 15-min interval between acquisitions. Interstudy rest and stress MBF and myocardial perfusion reserve (MPR) were measured for global myocardium and regionally for coronary territories and slices.

**Results:**

There was no significant difference in intrastudy repeated global rest MBF (0.65 ± 0.13 ml/g/min vs 0.62 ± 0.12 ml/g/min, *p* = 0.24, repeatability coefficient (RC) =24%) or stress (2.89 ± 0.56 ml/g/min vs 2.83 ± 0.64 ml/g/min, *p* = 0.41, RC = 29%) MBF. No significant difference was seen in interstudy repeatability for global rest MBF (0.64 ± 0.13 ml/g/min vs 0.64 ± 0.15 ml/g/min, *p* = 0.80, RC = 32%), stress MBF (2.71 ± 0.61 ml/g/min vs 2.55 ± 0.57 ml/g/min, *p* = 0.12, RC = 33%) or MPR (4.24 ± 0.69 vs 3.73 ± 0.76, *p* = 0.25, RC = 36%). Regional repeatability was good for stress (RC = 30–37%) and rest MBF (RC = 32–36%) but poorer for MPR (RC = 35–43%). Within subject coefficient of variation was 8% for rest and 11% for stress within the same study, and 11% for rest and 12% for stress between studies.

**Conclusions:**

Fully automated, inline, myocardial perfusion mapping by CMR shows good repeatability that is similar to the published PET literature. Both rest and stress MBF show better repeatability than MPR, particularly in regional analysis.

**Electronic supplementary material:**

The online version of this article (10.1186/s12968-018-0462-y) contains supplementary material, which is available to authorized users.

## Background

There is increasing evidence that revascularisation decisions in patients with coronary artery disease (CAD) should be based on objective measurements of ischaemia rather than visual assessment [1–3]. Positron emission tomography (PET) is the current reference standard for non-invasive quantification of myocardial blood flow (MBF) and detection of ischaemia. Quantitative perfusion cardiovascular magnetic resonance (CMR) offers an alternative to PET with the advantage of lack of ionising radiation, higher spatial resolution and the added value of providing comprehensive data on left ventricular size, function and scar within a single study. Quantification of MBF by CMR has been validated in several small scale studies against microspheres, PET and invasive fractional flow reserve (FFR) measurements [4–7]. However, the wider adoption of quantitative perfusion CMR has been limited by the need for time consuming, off line processing and poor repeatability [8]. Recently, a new motion corrected (MOCO) myocardial perfusion CMR method with Gadgetron-based automated in-line perfusion mapping has been proposed, allowing free breathing acquisition and pixel-wise quantification of MBF [9]. The method has been shown to have good correlation with PET in patients with stable CAD [10], but needs to proceed through a number of validation steps before it can be used clinically and as a research surrogate endpoint. In this study, the repeatability of myocardial perfusion mapping by CMR was assessed in healthy subjects.

## Methods

### Study population

Forty-two healthy subjects were recruited (23 female, median age 23 yrs., IQR 22–29 yrs). Exclusion criteria were known cardiovascular disease, hypertension, hyperlipidaemia, diabetes mellitus, smoking and any contraindication to CMR, adenosine or gadolinium based contrast agents.

### Study protocol

The 42 healthy subjects underwent repeat CMR studies in two groups to allow assessment of intrastudy and interstudy repeatability of rest and stress MBF as well as MPR.

**Fig. 1:**
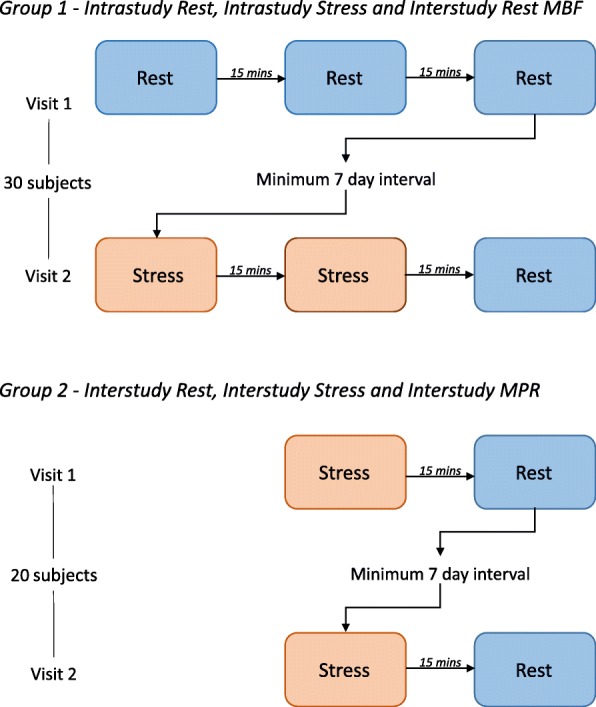
Study Protocol. Group one consisted of 30 volunteers, Group two included 8 volunteers from Group one, and an additional 12 healthy volunteers

- **Group 1**: 30 subjects underwent CMR studies on two separate visits. In visit 1, they had three rest perfusion scans and in visit 2, they had two stress and one rest perfusion scans. The acquisition of three rest scans in visit 1 allowed us to study the short-term repeatability of rest MBF with minimal physiological variation and to assess the effect of residual gadolinium (Gd) on repeated perfusion measurements. The fourth rest scan acquired in visit 2 was used to assess long-term variability of rest MBF. The two stress scans in visit 2 assessed short-term variability of stress MBF.
- **Group 2**: 20 subjects (8 from the first cohort and 12 additional subjects) had stress followed by rest perfusion scans in two separate visits to assess long-term repeatability of stress MBF and MPR (Fig. 1).

All CMR studies were carried out on a 3 T scanner (Magnetom Prisma, Siemens Healthineers, Erlangen, Germany). A minimum of 7 days was allowed between each visit (mean 41, SD 40 days). Subjects were advised to avoid caffeine for 24 h before each scan. Survey images were acquired, followed by vertical and horizontal long axis images to plan the short axis view for perfusion in three slice positions (basal, mid and apex).

Pharmacological stress was achieved with adenosine infusion at 140mcg/kg/min for a minimum of 3 min. The dose was increased to 175mcg/kg/min after 2 min if there was no symptomatic or haemodynamic response to adenosine. Subjects were monitored for symptoms and heart rate throughout the infusion, blood pressure and heart rate were recorded every 2 min during adenosine infusion. An intravenous bolus of 0.05 mmol/kg gadobutrol (Gadovist, Leverkusen, Germany) was administered at 5 ml/s followed by a 20 ml saline flush for each perfusion scan. A minimum gap of 15 min was maintained between each perfusion scan to allow for equilibrium of gadolinium kinetics from the previous series, and to ensure that the effects of adenosine had resolved.

Perfusion imaging used a dual sequence approach which employed separately optimized sequences for the myocardium and blood pool signals in order to avoid blood pool signal saturation. Full details of the myocardial perfusion sequence have been previously described [9]. Both sequences were electrocardiogram (ECG) triggered saturation recovery prepared. The sequence used for imaging the left ventricular (LV) blood pool to estimate the arterial input function (AIF) used a low flip angle FLASH low resolution protocol with 2 echos such that the echo times were short to minimize T2* losses at high concentration, and so that remaining T2* losses could be estimated and corrected. Parameters for this protocol were: flip angle 5 degrees, matrix 48 × 34, parallel imaging factor 2, TEs 0.76 and 1.76 ms, TR 2.45 ms, slice thickness 10 mm, saturation preparation used 6-pulse sequence, saturation delay TS 24 ms to k-space center, imaging duration 42 ms, total sequence duration 57 ms acquired immediately following the R-wave trigger. The myocardial imaging protocol in this study used a FLASH readout with typical imaging parameters: flip angle 14 degrees, spatial resolution 1.9 × 2.4 mm2, slice thickness 8.0 mm, TE/TR 1.0/2.1 ms, matrix size 192 × 111, field of view 360 × 270 mm2, parallel imaging acceleration factor 3, saturation recovery time (TS) 110 ms to center of k-space, trigger delay 72 ms, imaging duration 59 ms, saturation preparation used 5-pulse sequence, total duration including saturation 143 ms per slice, enabling acquisition of 3-slices plus AIF sequence in less than 500 ms permitting hear rates up to 120 bpm. Both AIF and myocardial imaging sequences included 3 measurements of proton density (PD) weighted images with flip angle of 5 degrees used for surface coil intensity correction. Slice spacing was varied on per patient basis to cover the LV.

In-line automatic reconstruction and post-processing were implemented within the Gadgetron software framework [9]. Images were motion corrected and then corrected for surface coil intensity variation based on the proton density weighted images. Signal intensity data were converted to Gd concentration (mmol/L) based on automatically generated look-up tables for the magnetization Bloch simulation. AIF data were extracted from the low-resolution Gd concentration images using automated segmentation of the LV cavity. MBF was calculated on a pixel-wise basis in the high-resolution images by blood tissue exchange (BTEX) model constrained deconvolution incorporating estimation of the delay time between bolus arrival in the LV cavity and the tissue of interest. Details of the reconstruction and processing including conversion to [Gd] concentration units, blood pool signal segmentation, and BTEX modeling are previously reported [9].

### Quantitative analysis

The in-line processing on the scanner console included the image reconstruction, respiratory motion correction, LV blood pool segmentation, conversion of signals to [Gd] concentration units, and quantitative tissue mapping. These steps were fully automatic. For a protocol with AIF plus 3-slices acquired for 60 measurements, the processing time was < 3 min.

**Fig. 2:**
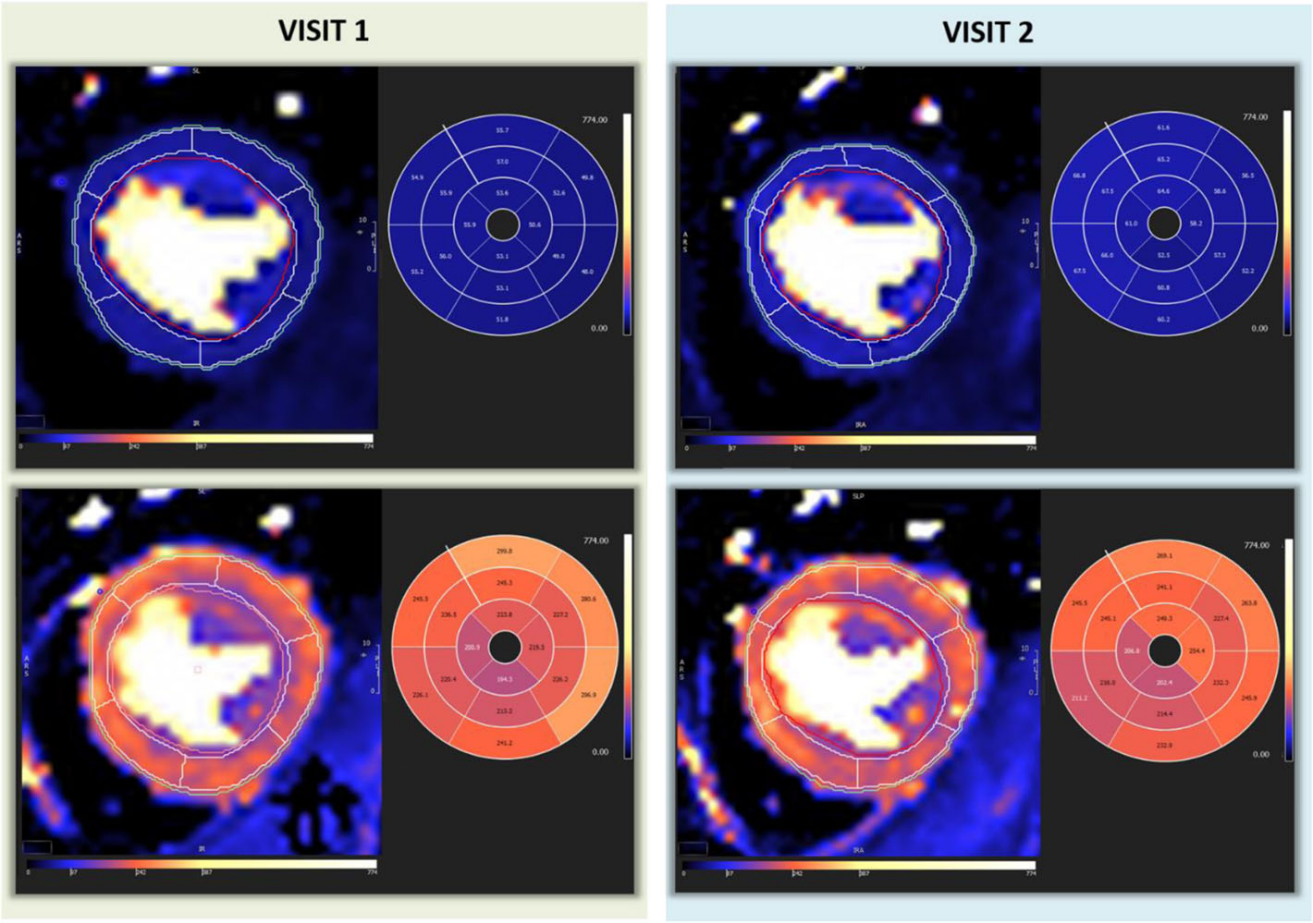
Rest and Stress MBF maps from visit 1 and visit 2 for the same subject. Values are displayed as ml/100 g/min

The analysis of the quantitative maps was performed off-line on a separate workstation using CVi 42 software (Circle Cardiovascular Imaging, Calgary, Canada). This process was performed manually by tracing endo- and epi-cardial contours for each slice and marking RV insertion points; a 16-segment American Heart Association (AHA) model was then applied [11]. In order to minimise partial volume effect, a 10% offset was applied to endocardial and epicardial borders (Fig. 2). MBF was recorded for each of the 16 segments. Where the LV outflow tract was included in the basal slice, or partial volume effect meant segments were too thin to contour, these segments were excluded from further analysis. Analysis time was less than 5 min per set of perfusion maps.

Segmental values were averaged to give values for slice, coronary territory and global MBF. Coronary territories consisted of: left anterior descending coronary artery (LAD) - segments 1, 2, 7, 8, 13 and 14, circumflex coronary artery (Cx) - segments 5, 6, 11, 12 and 16, and right coronary artery (RCA) – segments 3, 4, 9, 10 and 15.

Correlation of MBF with HR and RPP were analysed, and where significant correlation was present, MBF values were corrected. Values for resting MBF were corrected for heart rate (HR) by dividing by scan heart rate and multiplying by the mean resting HR (62 bpm) among all subjects. Interstudy repeatability was analysed on a regional basis for slices (basal, mid and apical) and coronary territories (LAD, Cx and RCA). MPR was calculated as a ratio of stress MBF:rest MBF.

### Reproducibility of analysis

Intra- and inter-observer variability were assessed by repeating the analysis of 10 subject data sets for stress and rest after 1 month, by the same observer (LB) and by a second observer (SO). The second observer was blinded to the previous results.

### Statistical analysis

Analysis was performed using SPSS 23 (International Business Machines, Armonk, New York, USA). Normality of data distribution was assessed using Shapiro-Wilk test. Data are presented as mean ± standard deviation (SD). Repeatability was assessed using a wide range of methods to facilitate comparison with the inconsistent methods in the published literature. The three intrastudy rest scans were compared using repeated measures analysis of variance (ANOVA) with Bonferroni adjustment for post-hoc analysis. All other repeated mean MBF and inter- and intra- observer variability were compared using paired t tests. Coefficient of variation (CV) was calculated using the root mean square method [12]. Reproducibility coefficient (RC) was calculated as 1.96*SD of difference and given as a percentage of the total mean and used to demonstrate bias and accuracy with Bland Altman plots. Reliability was assessed using intraclass correlation coefficient (ICC). All statistical tests were two-tailed and a *p* value < 0.05 was considered significant.

## Results

All subjects tolerated repeated CMR scans and adenosine stress well. One subject did not attend the second visit for assessment of intrastudy rest repeatability, another was not included in intrastudy stress analysis due to triggering problems causing artefact on the MBF maps on one stress scan. One result was excluded from analysis of repeat stress MBF or MPR due to lack of stress response on one visit, confirmed by lack of symptoms despite increased adenosine dose, haemodynamic response and splenic switch off. One result was excluded from comparison of MPR due to severe artefact on rest perfusion maps.

### Intrastudy repeatability

Twenty-nine studies were analysed for intrastudy repeatability of resting and hyperaemic MBF from the two separate visits of Group 1.

**Fig. 3:**
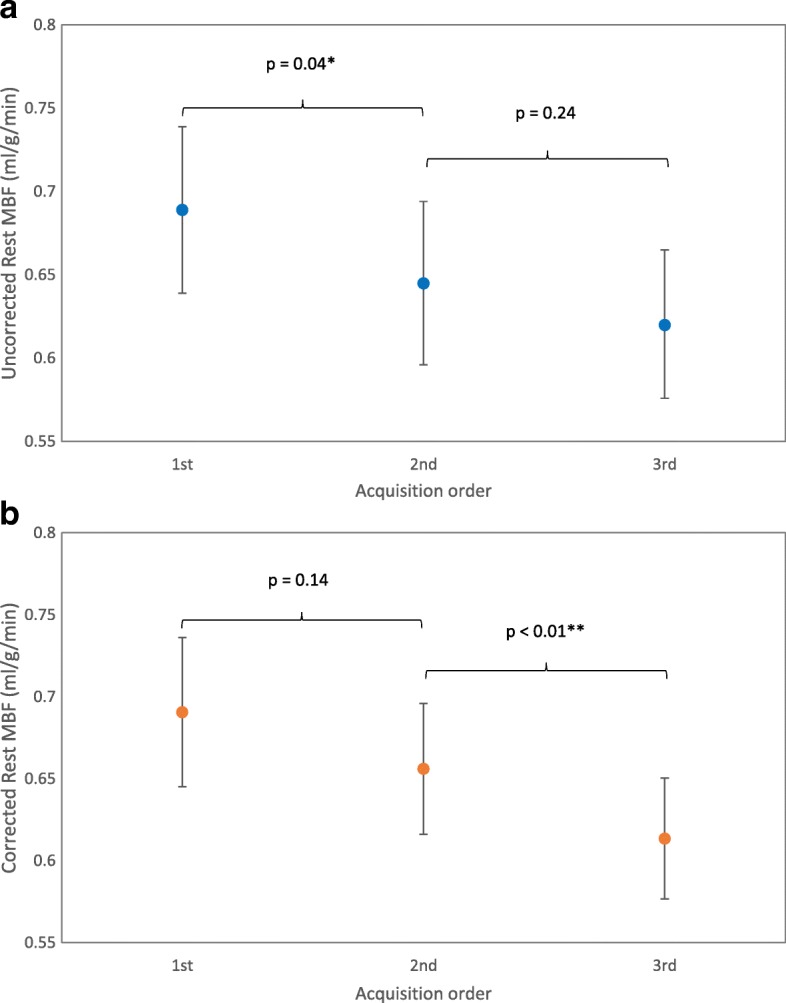
Global resting myocardial blood flow (MBF) on repeat throughout the same visit. A – uncorrected values, B – corrected values (mean and 95% confidence interval of the mean)

**Fig. 4:**
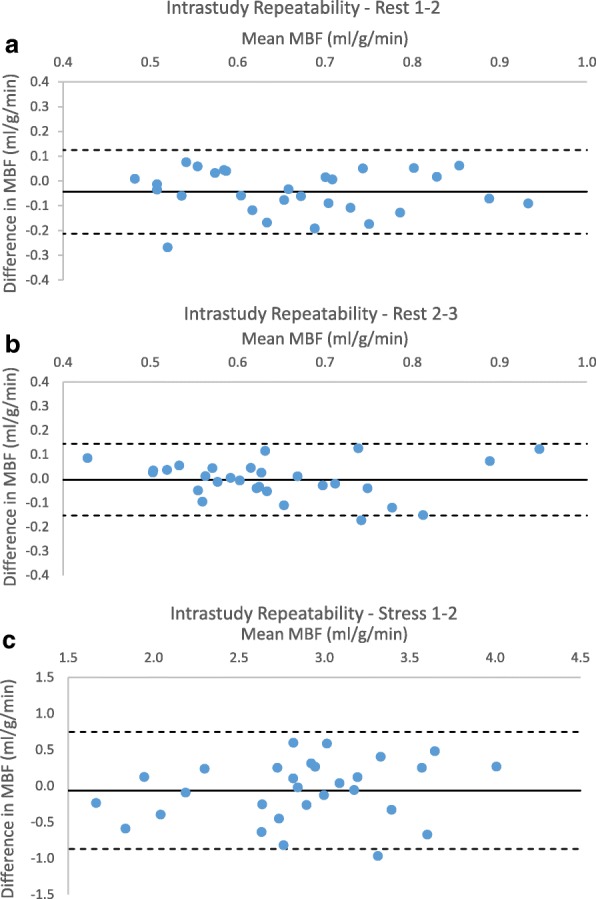
Intrastudy myocardial blood flow (MBF) repeatability (**a**) Rest 1–2 (**b**) Rest 2–3 (**c**) Stress 1–2

Mean global MBF at rest was 0.69 ± 0.13 ml/g/min, 0.65 ± 0.13 ml/g/min and 0.62 ± 0.12 ml/g/min for scans 1, 2 and 3 respectively (Table 1). There was a significant difference in mean MBF on the first rest scan compared to both the second (*p* = 0.01) and third (*p* = 0.001). There was no significant difference between the second and third scans (Fig. 3). Coefficient of variation was 11–12% between the first scan and repeats, and 8% between second and third scans with good reliability (ICC = 0.8, RC 24%). Assessment of repeatability with Bland-Altman plots (Fig. 4) showed a bias of − 0.03 ml/g/min (3.9% of the mean).

**Table 1:** Intrastudy repeatability of global MBF measurements

	Test 1 ml/g/min		Test 2 ml/g/min		Difference in mean ml/g/min		*p*	RC	RC (%)	CV (%)	ICC
Rest							< 0.01				
Rest 1 - Rest 2	0.69	±0.13	0.65	±0.13	−0.04	±0.09	0.04	0.19	28.5	10.9	0.73
Rest 1 - Rest 3	0.69	±0.13	0.62	±0.12	−0.07	±0.10	0.02	0.23	35.2	11.9	0.58
Rest 2 - Rest 3	0.65	±0.13	0.62	±0.12	−0.03	±0.07	0.24	0.15	23.8	7.93	0.80
Stress											
Stress 1- Stress 2	2.89	±0.56	2.83	±0.64	−0.06	±0.42	0.41	0.82	28.5	10.6	0.76

*p* – from repeated measures ANOVA and level of significance using Bonferroni post-hoc analysis for rest data, Student’s T-test for stress values

*RC* repeatability coefficient, *RC (%)*repeatability coefficient as percentage of the mean, *CV* within subject coefficient of variation, *ICC* intraclass correlation coefficient

Resting MBF correlated with HR (*r* = 0.49, *p* < 0.01), therefore MBF corrected for HR was also analysed (Table 2). A significant difference was still present between the groups (*p* < 0.01) and the decrease with sequential scans remained (0.69 ml/g/min, 0.66 ml/g/min and 0.61 ml/g/min for scans 1, 2 and 3). A significant difference was seen between scan 3 and the other two results (*p* < 0.01). Whilst the level of significance differed when corrected for heart rate, both sets of values showed a trend to decrease with repeated measurement (Fig. 3).

**Table 2:** Global rest myocardial blood flow (MBF) corrected for heart rate (HR)

	Test 1 ml/g/min		Test 2 ml/g/min		Difference in mean ml/g/min		*p*	RC	RC (%)	CV (%)	ICC
Rest							< 0.01				
Rest 1 - Rest 2	0.69	±0.12	0.66	±0.10	−0.03	±0.09	0.14	0.18	27.3	9.9	0.66
Rest 1 - Rest 3	0.69	±0.12	0.61	±0.10	−0.07	±0.07	< 0.01	0.20	30.3	10.5	0.62
Rest 2 - Rest 3	0.66	±0.10	0.61	±0.10	−0.04	±0.06	< 0.01	0.15	23.7	8.3	0.72

p – from repeated measures ANOVA and Bonferroni post-hoc analysis

*RC* repeatability coefficient, *RC (%)*repeatability coefficient as percentage of the mean, *CV* within subject coefficient of variation, *ICC* intraclass correlation coefficient

Stress MBF showed no significant difference between the two repeat acquisitions in visit 2 (mean difference − 0.06 ± 0.42, *p* = 0.41). Within subject coefficient of variation was 11% with good correlation and repeatability (ICC 0.76, RC 29%). One value was outside the limits of agreement on assessment with Bland-Altman plots, with a bias of 2.2% of the mean (Fig. 4). Stress RPP was comparable between both studies (11,202 ± 2188 vs 10,858 ± 1877, *p* = 0.09) as was the percentage increase in HR (47.3 ± 18.8% vs 44.4 ± 18.4%, *p* = 0.24) and RPP (51.1 ± 21.7% vs 46.9 ± 22.1%, *p* = 0.14).

### Interstudy repeatability

#### Global perfusion analysis

**Fig. 5:**
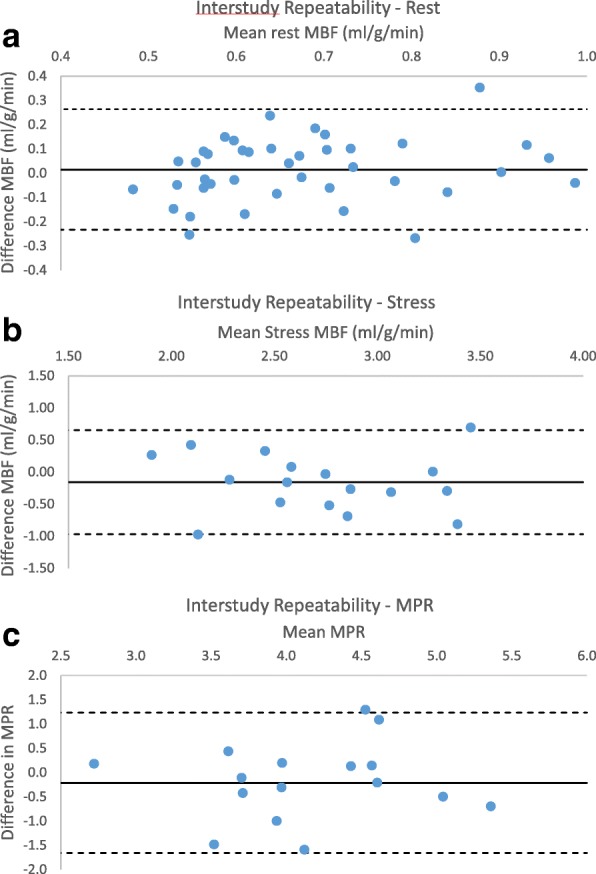
Interstudy repeatability (**a**) Rest MBF (**b**) Stress (**c**) myocardial perfusion reserve (MPR)

A total of 41 studies were analysed for interstudy repeatability of resting MBF with an average gap of 27 days between scans in visits 1 and 2. No significant difference was seen in MBF between scans (mean difference 0.004 ± 0.1 ml/g/min, *p* = 0.8) (Table 3). Within subject coefficient of variation was 11%, RC 32% and bias was < 1% of the mean (Fig. 5).

**Table 3:** Interstudy repeatability of MBF measurements – by slice and coronary artery territory

	Test 1 ml/g/min		Test 2 ml/g/min		Difference in mean ml/g/min		p	RC	RC (%)	CV (%)	ICC
Rest											
Global	0.64	±0.13	0.64	±0.15	0.004	±0.10	0.8	0.20	31.5	11.3	0.74
Basal	0.66	±0.14	0.67	±0.16	0.015	±0.13	0.46	0.25	37.7	13.6	0.65
Mid	0.64	±0.14	0.64	±0.15	−0.003	±0.10	0.87	0.20	31.0	11.2	0.76
Apex	0.60	±0.13	0.60	±0.15	−0.003	±0.10	0.86	0.20	33.4	11.5	0.73
*P*	0.13		0.089								
LAD	0.69	±0.16	0.68	±0.16	−0.003	±0.11	0.86	0.22	32.2	11.7	0.75
Cx	0.60	±0.13	0.60	±0.14	0.005	±0.11	0.74	0.20	34.0	11.8	0.70
RCA	0.61	±0.12	0.62	±0.15	0.01	±0.11	0.59	0.22	35.6	12.6	0.66
*P*	0.01*		0.04*								
Stress											
Global	2.71	±0.61	2.55	±0.57	−0.161	±0.43	0.12	0.87	33.1	12.2	0.72
Basal	3.01	±0.80	2.80	±0.74	−0.209	±0.51	0.09	1.05	36.1	13.2	0.76
Mid	2.48	±0.55	2.39	±0.51	−0.092	±0.44	0.38	0.86	35.4	13.4	0.66
Apex	2.62	±0.65	2.42	±0.56	−0.201	±0.62	0.17	1.24	49.2	16.3	0.48
*P*	0.05		0.07								
LAD	2.79	±0.61	2.62	±0.55	−0.167	±0.39	0.08	0.82	30.3	10.8	0.75
Cx	2.69	±0.61	2.51	±0.66	−0.180	±0.47	0.12	0.97	37.3	15.4	0.71
RCA	2.53	±0.61	2.44	±0.56	−0.096	±0.47	0.39	0.92	37.0	13.6	0.68
*P*	0.442		0.629								
MPR											
Global	4.24	±0.69	3.73	±0.76	−0.214	±0.76	0.25	1.46	36.4	13.3	0.46
Basal	4.53	±0.90	4.27	±1.00	−0.262	±0.89	0.23	1.78	40.4	15.3	0.55
Mid	3.84	±0.70	3.73	±0.66	−0.113	±0.83	0.57	1.60	42.4	15.5	0.26
Apex	4.42	±0.90	4.12	±0.76	−0.293	±1.04	0.25	2.06	48.1	16.0	0.30
*p*	0.047*		0.130								
LAD	3.96	±0.58	3.83	±0.81	−0.128	±0.71	0.45	1.35	35.3	13.1	0.50
Cx	4.47	±0.93	4.14	±0.93	−0.325	±0.86	0.13	1.76	40.8	15.7	0.54
RCA	4.06	±0.76	3.91	±0.66	−0.150	±0.89	0.49	1.72	43.3	15.7	0.23
*p*	0.118		0.480								

*RC* repeatability coefficient, *RC (%)*repeatability coefficient as percentage of the mean, *CV* within subject coefficient of variation, *ICC* intraclass correlation coefficient

p – level of significance using Student’s T-test

*=significant at *p* < 0.05

Nineteen pairs of scans were analysed for interstudy stress MBF and 18 for interstudy MPR in Group 2. The percentage increase in HR (52.1 ± 26.6% vs 50.4 ± 23.4%, *p* = 0.7) and RPP (56.6 ± 32.7% vs 52.1 ± 26.6%, *p* = 0.11) between rest and stress scans showed no significant difference between visits. Adenosine stress MBF had good repeatability with ICC 0.72 and RC 33%. CV was 12% and bias was − 6% of the mean. Repeat MPR had a CV of 13.3 with no significant difference between the two measurements. Weaker correlation was seen compared to stress and rest, although this remained significant (ICC 0.46, *p* < 0.01).

#### Regional perfusion analysis

At rest there was no significant difference between slices (*p* = 0.13 and 0.09 for first and second scans) (Table 3). No significant difference was seen in individual slices between scans and all showed good repeatability and correlation (*p* < 0.01 in all slices) (Additional file 1: Figure S1). Coefficient of variation was 13.6, 11.2 and 11.5% for basal, mid and apical slices respectively.

Mean MBF at stress was 3.01 ± 0.8 ml/g/min, 2.48 ± 0.55 ml/g/min and 2.62 ± 0.65 ml/g/min on the first visit and 2.8 ± 0.74 ml/g/min, 2.39 ± 0.51 ml/g/min and 2.42 ± 0.56 ml/g/min in basal, mid and apical slices respectively. No significant difference was seen between scans for any slice. The apical slice exhibited the lowest repeatability, ICC 0.46, RC 49% and within subject coefficient of variation 16%. Good correlation was seen in all slices (Additional file 1: Figure S1).

No significant difference was seen in mean MPR between visits for any slice, RCs were 40, 42 and 48% and CVs 15, 15 and 16% for basal mid and apical slices.

Coronary territory flows were significantly different between vessel territories at rest on both visits. MBF in the LAD was higher than the Cx territory on both visits (mean difference 0.09 ml/g/min, *p* = 0.01 on the first scan, and 0.08 ml/g/min, *p* = 0.04 on the second). There was good correlation within all coronary territories between scans (Additional file 2: Figure S2). All territories showed similar ICC (0.66–0.75) and repeatability coefficients (32–36%). CVs were very similar between territories (11.7, 11.8 and 12.6%).

No significant difference was seen between coronary territories in stress MBF, or in calculated MPR at either scan. All coronary territories showed good repeatability and correlation between scans. Coefficients of variation ranged between 10.8 and 15.4%, being highest in the circumflex territory.

No significant difference was seen in MPR in any coronary territory between visits. The LAD and Cx territories showed acceptable correlation and repeatability coefficients (LAD: ICC 0.5, RC 35%, Cx: ICC 0.54, RC 41%). The RCA territory did not show significant correlation between visits, ICC = 0.23.

#### Interobserver and Intraobserver repeatability

Ten sets of perfusion maps were assessed for intraobserver variability at a minimum gap of 4 weeks between analysis, and for interobserver variability. There was excellent agreement for all measurements (Table 4). The highest coefficients of variation were seen in the apical slice.

**Table 4:** Intra and inter-observer MBF reproducibility

	Intra-observer		Inter-observer	
	CV (%)	ICC	CV (%)	ICC
Rest				
Global	0.7	0.999	0.8	0.999
Basal	1.1	0.998	1.2	0.998
Mid	1.0	0.999	0.6	0.999
Apical	3.0	0.990	3.7	0.980
LAD	1.1	0.997	1.3	0.996
Cx	1.4	0.997	1.3	0.995
RCA	1.8	0.996	1.8	0.991
Stress				
Global	2.0	0.995	2.4	0.995
Basal	2.0	0.996	3.0	0.993
Mid	2.6	0.999	3.3	0.994
Apical	6.3	0.963	8.0	0.959
LAD	2.8	0.99	2.6	0.99
Cx	2.5	0.992	4.3	0.986
RCA	1.9	0.992	1.9	0.993

*CV* coefficient of variation, *ICC* intraclass correlation coefficient

## Discussion

The main findings of this study are 1) Gadgetron in-line myocardial perfusion mapping by CMR has good short and long-term repeatability, 2) regional assessment of coronary artery territory MBF has good repeatability, 3) MPR is a less reproducible method of assessment than MBF, particularly for regional assessment.

### Global perfusion analysis

We have shown good repeatability of global stress and rest MBF for automated perfusion mapping with CMR, both within one scan and with an interval between scans. Our results are consistent with previous studies, using both invasive and non-invasive estimates of MBF and MPR.

Invasive assessment of coronary flow reserve has a coefficient of variation of 19% on repeat within minutes [13]. Table 5 contains a summary of the published literature on repeatability of non-invasive MBF measurement. PET repeatability in the published literature has ranged from RCs of 18–35% for resting MBF and 18–41% for stress MBF [14–17]. The largest study of test-retest repeatability in PET to date involved 120 volunteers who underwent serial stress or rest scans. Rest MBF CVs were 10.7% within test and 21.1% between tests, and stress MBF CVs were 9.6–10.6 and 19–21% [18]. As in most of the published literature, short-term repeatability had lower coefficients of variation and repeatability than delayed repeatability. In a recent study to assess the optimal kinetic model for repeatability in PET the best results gave a combined repeatability coefficient of 15.8% for stress and rest [19].

**Table 5:** Summary of literature on MBF repeatability

				Rest				Stress				MPR			
	Author	Year	n	T test	r/ICC	RC (%)	CV (%)	T test	r/ICC	RC (%)	CV (%)	T test	r/ICC	RC (%)	CV (%)
*Immediate (intrastudy)*															
PET	Nitzsche [30]	1996	15	0.33	0.99	33		0.16	0.97	13					
	Kaufmann [14]	1999	21	ns		18		ns		25		ns		33	
	Wyss [23]	2003	11	ns	0.77	21			0.77	27		ns	0.74	35	
	Schindler [31]	2007	20		0.72	29			0.76	20					
	Manabe [15]	2009	15	0.31		22		0.81		27		0.53		37	
	Kitkungvan [18]	2017	120	0.93			11	0.74			10				
	Ocneanu [19]	2017	12				21				15				
CMR	Keith^a^ [32]	2017	10			53	13								
	This study			0.08	0.8	24	8	0.41	0.76	29	11				
*Delayed (interstudy)*															
PET	Nagamachi [16]	1996	30	ns	0.63	31		ns	0.69	18				20	
	Schindler [31]	2007	20		0.75	30			0.71	23					
	Sdringola [17]	2011	48	*p* < 0.05	0.68	35		ns	0.53	34		ns	0.47	38	
	Johnson [24]	2015	50	0.46		41		0.13		34		0.29		34	
	Kitkungvan [33]	2017	19	0.13				0.94			17	0.26			20
	Kitkungvan [18]	2017	120	0.13			21	0.81			19				
CMR	Jerosch-Herold [20]	2008	30	0.001		30		0.11		41					
	Larghat^a^ [8]	2013	11	0.2		45	20	0.61		73	40	0.11		69	35
	Likhite [21]	2016	10		0.77								0.88		
	Keith^a^ [32]	2017	10			61	16								
	This study			0.8	0.74	32	11	0.12	0.72	33	12	0.25	0.44	36	13

*ns* not significant (*p* value not reported), *r* Pearson correlation coefficient, *RC* reproducibility coefficient (% of mean), *CV* coefficient of variation

^a^Repeatability data is given for single mid-ventricular slice, all other studies, data is for global myocardium, averaged from multiple slices. Where RC was not published, but sufficient data was provided, this has been calculated using 1.96*SD of difference. Similarly, all RC are given as % for ease of comparison

There are few CMR studies of repeatability. The largest, a subset of the Multi-Ethnic Study of Atherosclerosis (MESA) study, included 30 patients with an interval of almost a year between scans [20], and reported repeatability coefficients of 30 and 41% for rest and stress MBF respectively, similar to values in PET studies. Another small sample of 10 patients showed good correlation for interstudy repeatability at rest (*r* = 0.77) and stress (*r* = 0.9) and CVs of 23% at rest and 20% for stress, in keeping with previous PET literature [21]. Other CMR studies have shown poorer repeatability than PET; a more recent, smaller study of 11 subjects [8] showed repeatability coefficients of 45 and 73%, with coefficients of variation of 20 and 40% for rest and stress respectively.

Our data for automated in line perfusion CMR mapping fit well within this published data and are comparable to the best results achieved with PET. Compared with most CMR studies, the repeatability in the current study was better for global MBF at both rest and stress, and for both short and long-term repeatability.

### Regional assessment

All coronary territories showed good repeatability for rest and stress MBF. Higher repeatability coefficients and coefficients of variation were seen than for global values, consistent with the limited published literature in CMR and some of the PET literature. These findings likely reflect the smaller volume of myocardium assessed in regional assessment. Early CMR studies of repeatability have included only the mid slice but have shown regional repeatability to be higher than global (RC 28% vs 21%) [22]. The PET literature is inconsistent regarding regional vs global repeatability. One PET study examined coronary territory MBF repeatability in 30 patients and reported similar repeatability coefficients to global flow at stress (RC 18% for global perfusion, 18–24% for regional perfusion) and at rest (RC 31% for global and 26–46% for regional perfusion). A further study of 48 subjects showed comparable values for global perfusion vs regional perfusion (quadrants), with regional rest values of RC 33–41% and stress 33–38% vs global values 35 and 34% [17]. Other PET studies have shown worse repeatability in regional assessment compared to global values. A study of 21 subjects assessing repeatability of ^15^O PET had regional repeatability coefficients of 22–46% at rest and 41–59% at stress vs 18 and 25% globally [14]. A smaller study of 11 showed regional repeatability coefficients of 38–55% at rest and 55–70% at stress vs 21 and 27% globally [23]. Numerically the regional variability in our study was similar to or lower than in most PET studies. All coronary territories showed good correlation and repeatability with RCs of 32–36% at rest and 30–37% at stress.

In addition to coronary territories, we also compared variability between the three acquired slices. At stress the apical slice had the lowest ICC, showing moderate reliability (ICC = 0.48, *p* = 0.02). It also showed the highest inter- and intra-observer variability, with higher coefficients of variation than other regions. This underlines the difficulty in assessment and quantification of the most apical slice, due to the small area of myocardium available for analysis and the larger partial volume effect due to the conical shape of the apex, exacerbated at stress by increased cardiac motion.

### MPR assessment

We found that the repeatability of CMR MPR was lower than that of MBF, in particular for the analysis of coronary territories. Previous studies have shown similar values for repeatability of global MPR to resting and stress MBF, with PET values of multiple studies within the range of 33–38%, in keeping with our results [14, 15, 17, 23, 24]. A study of 30 subjects showed a repeatability coefficient of 20% for MPR; however, these results included some studies repeated within the same day, which may account for the lower values. Data on inter-test reproducibility for MPR in CMR is limited, with a single study of 11 subjects showing a RC of 69%, higher than PET data and those from this study [8].

Where regional MPR has been compared to global values for repeatability, some studies have shown markedly worse repeatability. One study showed regional RC of 68–82% vs 33% for global values [14] another showed an increase from 35% for to 67–96% [23]. Others have shown a small rise or comparable RC in a similar pattern to our data. The largest study, with 48 paired studies showed a RC of 38% globally with a maximum of 43% in the lateral wall [17].

The lack of significant correlation in repeated results both for apical and mid slices and the RCA territories supports the use of stress MBF rather than MPR for clinical assessment where regional differences are diagnostically important. It is known that stress MBF correlates with the severity of stenosis in CAD [25], therefore the better reliability we have demonstrated for stress MBF compared with MPR would support its use for this important diagnostic decision.

### Variation in resting MBF

We have shown variation in resting MBF on short-term repeat, within the same scan. A trend for rest MBF to decrease with serial measurement was not removed by correction for heart rate. Whilst the level of significance in this difference altered when MBF was corrected for HR, there remained a trend for decrease with sequential repeat (Fig. 3).

Published literature has not shown this decrease in MBF during the same scan. Two studies have shown a decrease in resting MBF on repeat assessment with a longer interval between imaging. In a study of healthy subjects with no risk factors, a significant decrease of 0.05 ± 0.13 ml/g/min (*p* < 0.05) was seen with a median interval of 22 days between scans [17]. This effect was not present in those with cardiovascular risk factors or in stress perfusion. The MESA study produced a similar finding with a decrease in resting MBF of 0.1 ml/g/min, *p* = 0.001 over a longer time interval (mean 334 days) [20]. This change was also accompanied by a significant change in heart rate, which was proposed to account for the drop.

Although some of these results differ from those in our study and the intervals between scans were different from in our study, they do provide more evidence of the susceptibility of rest MBF to change and physiological variation. Our study is the first to report more than 2 repeated measurements within one scanning session. The decrease seen within our study may be caused by an element of anxiety present at the beginning of the CMR scan in the subjects examined. In addition to a rise in heart rate and blood pressure, stress is associated with vasodilation of the coronary microvasculature in healthy volunteers [26]. Following correction for heart rate, the decrease in MBF remained, suggesting that vasodilation rather than cardiac work is the cause of the higher values on the first scans. This assumption is supported by the lack of difference between sequential stress perfusion, where maximal vasodilatation is induced so that differences in anxiety have no additional impact. The absence of significant difference in sequential stress scans also supports that this change is likely to be physiological rather than an effect of residual Gd from the previous series. The lack of significant difference between the second perfusion acquisition and subsequent assessment is reassuring clinically, as this would imply that stress followed by rest perfusion assessment, the most commonly used protocol, would produce repeatable values for both rest and stress.

### In-line perfusion mapping

The development of Gadgetron automated inline perfusion mapping overcomes one of the main previous limitations of quantitative perfusion CMR by removing the previously time-consuming analysis and the need for specialist knowledge. We have shown that this technique provides measurements of MBF with a repeatability that is comparable to the reference method PET and that is at least as good as previous, manual, CMR analysis methods. A recent study has shown that CMR perfusion mapping yields similar MBF values at rest and vasodilator stress as ammonia PET in patients with stable CAD [10]. Assessment using perfusion maps allows objective assessment of MBF, providing simpler and faster analysis and may have clinical advantages of detecting disease with global decrease in MBF such as microvascular and multivessel disease.

### Study limitations

Our data are influenced by physiological variation as well as variation within the model and analysis. While we aimed to minimise physiological variation as much as possible, some effects may not have been controlled for. Caffeine has been demonstrated to affect coronary vasomotor tone at rest [27] and adenosine stress perfusion CMR [28]. Although we advised our volunteers to avoid caffeine for 24 h prior to the scan, previous studies have demonstrated that up to 20% may still have detectable caffeine levels [29]. In addition, dosing of adenosine was determined clinically according to symptoms and response rather than a direct repeat from the previous scan. This would mimic clinical practice, and any difference in doses required may result in physiological variants in response. We can be confident that adequate stress was achieved; having seen appropriate increases in HR and RPP together with symptomatic response, however, the degree of hyperaemia may vary from maximal and account for the increased variation seen in stress MBF. All studies were performed using a FLASH perfusion sequence at 3 T, results for MBF may vary using other sequences or field strengths.

## Conclusion

Quantitative perfusion CMR using automated perfusion mapping achieves estimates of MBF and MPR with repeatability similar to the reference standard method PET. In this study rest and stress MBF, rather than MPR were a more reproducible assessment, particularly in regional analysis. The degree of physiological variation emphasises the importance in establishing normal ranges to allow for accurate diagnostic use.

## Additional files

Additional file 1: **Figure S1.** Correlation by slice (A) rest (B) stress. Trend line represents line of perfect fit. (DOCX 119 kb)

Additional file 2: **Figure S2.** Correlation by coronary territory (A) rest (B) stress. Trend line represents line of perfect fit. (DOCX 116 kb)

## Abbreviations

AHA: American Heart Association
AIF: arterial input function
ANOVA: analysis of variance
BP: blood pressure
BTEX: blood tissue exchange
CAD: coronary artery disease
CFR: coronary flow reserve
CMR: cardiovascular magnetic resonance
CV: coefficient of variation
Cx: circumflex
ECG: Electrocardiogram
FFR: fractional flow reserve
Gd: gadolinium
HR: heart rate
ICC: intraclass correlation coefficient
IQR: interquartile range
LAD: left anterior descending
MBF: myocardial blood flow
MOCO: motion corrected
MPR: myocardial perfusion reserve
PD: Proton density
PET: Positron emission tomography
RC: repeatability coefficient
RCA: right coronary artery
RPP: rate pressure product
SD: standard deviation
TE: Echo time
TR: Repetition time
TS: Saturation recovery time
