# Supplementary figures and images for: Fully automated, inline quantification of myocardial blood flow with cardiovascular magnetic resonance: repeatability of measurements in healthy subjects
Source: J Cardiovasc Magn Reson. 2018 Jul 9;20:48. doi: 10.1186/s12968-018-0462-y (PMC6036695; doi:10.1186/s12968-018-0462-y)

A


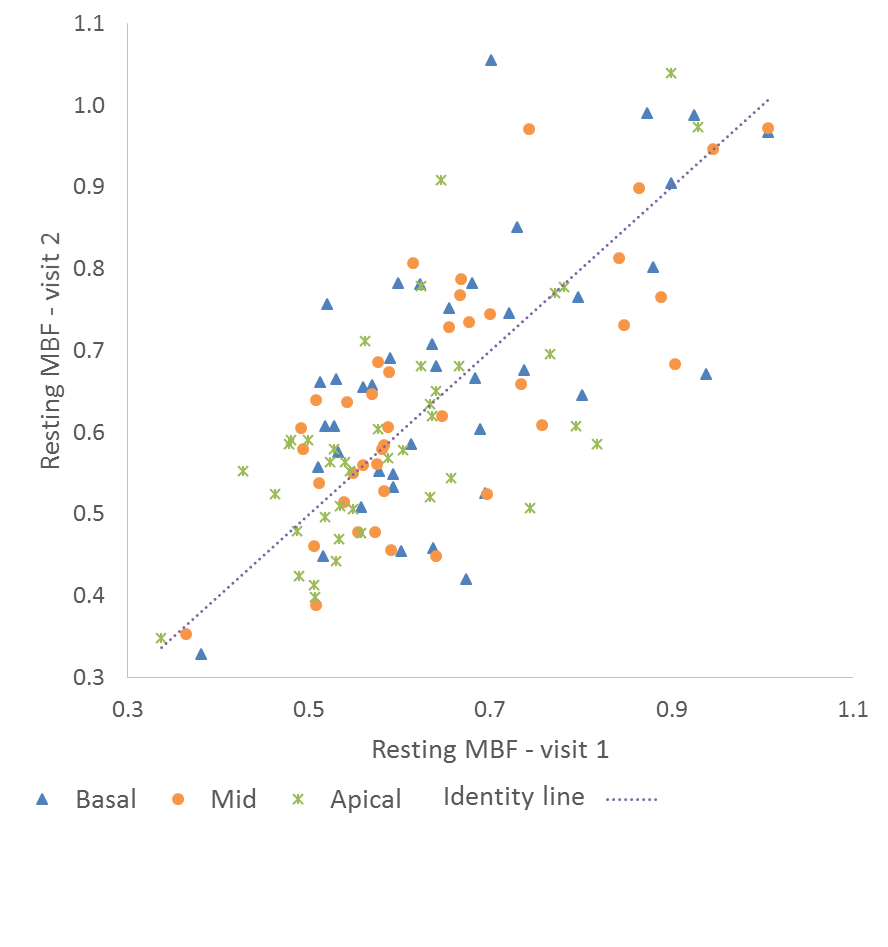


B


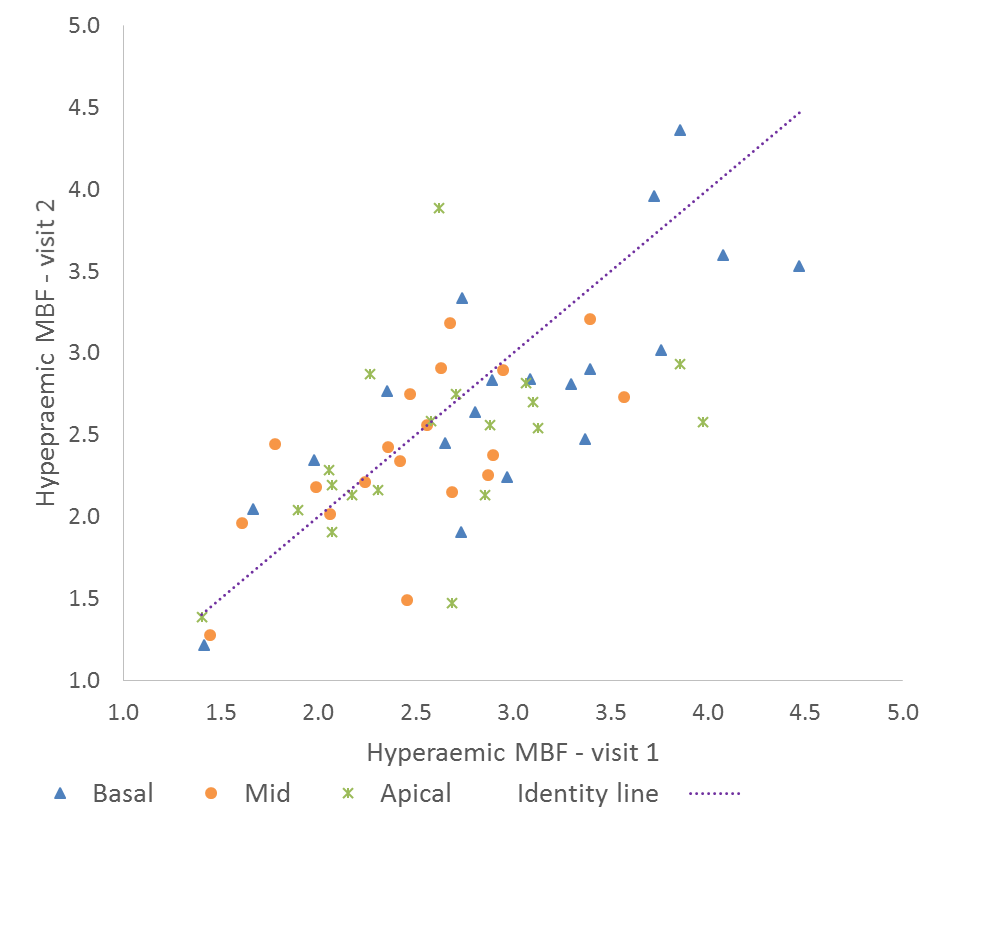

Supplement: Supplementary file 1 — Figure S1. Correlation by slice (A) rest (B) stress. Trend line represents line of perfect fit. (DOCX 119 kb) [file 12968_2018_462_MOESM1_ESM.docx]

A


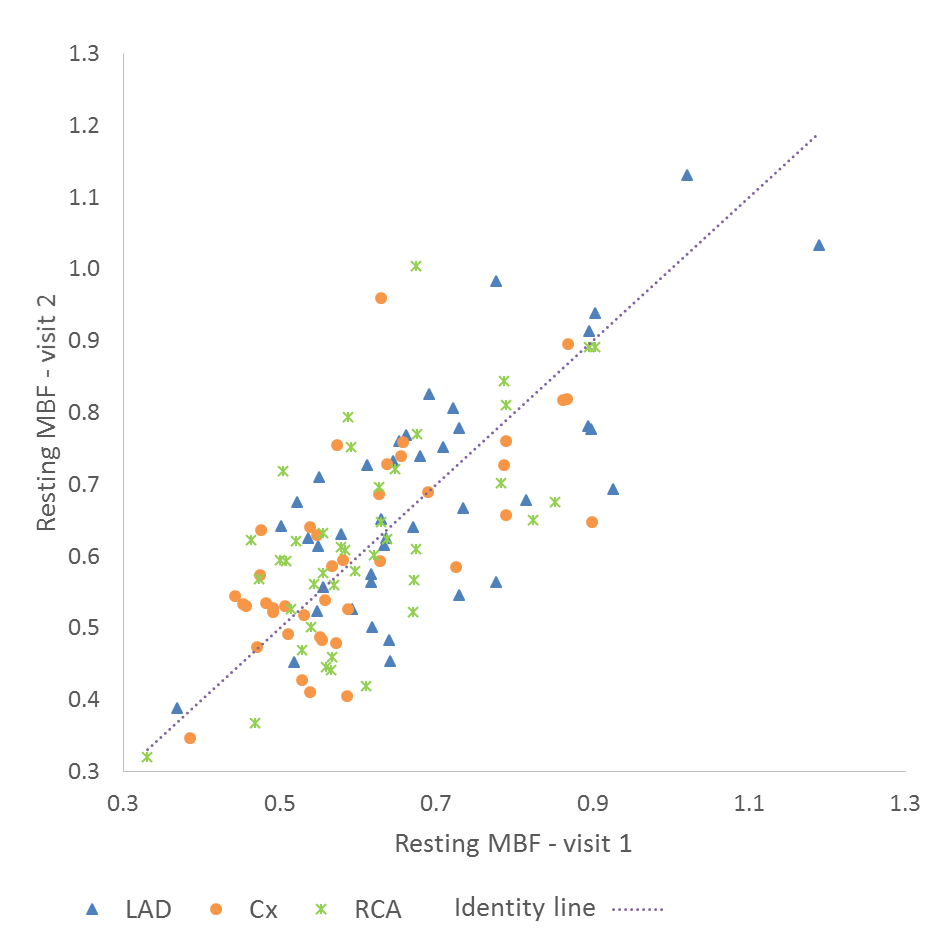


B


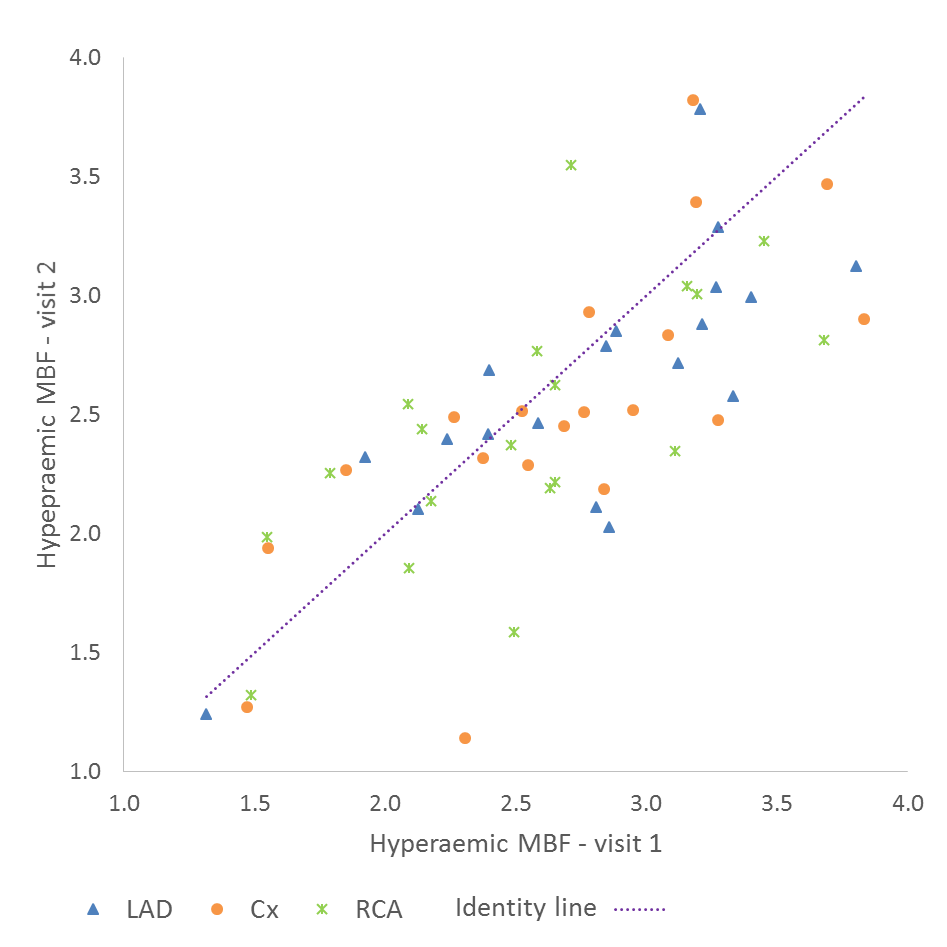

Supplement: Supplementary file 2 — Figure S2. Correlation by coronary territory (A) rest (B) stress. Trend line represents line of perfect fit. (DOCX 116 kb) [file 12968_2018_462_MOESM2_ESM.docx]
